# Association between gastric cancer and the intake of different types of iron and meats

**DOI:** 10.1186/s40795-023-00688-y

**Published:** 2023-03-21

**Authors:** Saba Narmcheshm, Fatemeh Toorang, Bahareh Sasanfar, Maryam hadji, Sahar Rostami, Kazem Zendehdel

**Affiliations:** 1 Department of Community Nutrition, School of Nutritional Sciences and Dietetics, Tehran University of Medical Sciences, Tehran, Iran; 2 Cancer Research Center, Cancer Institute, Tehran University of Medical Sciences, Tehran, Islamic Republic of Iran; 3 Department of Nutrition, School of Public Health, Shahid Sadoughi University of Medical Sciences, Yazd, Iran; 4 Health Sciences Unit, Faculty of Social Sciences, Tampere University, Tampere, Finland; 5 Department of Anatomy, School of Medicine, Tehran University of Medical Sciences, Tehran, Iran; 6 Cancer Biology Research Center, Cancer Institute, Tehran University of Medical Sciences, Tehran, Islamic Republic of Iran

**Keywords:** Heme Iron, Non-Heme Iron, Meat Intake, Stomach Neoplasms

## Abstract

**Background:**

Heme and non-heme irons are two forms of iron in the diet. Few studies have evaluated the association between heme iron intake and the risk of gastric cancer (GC). We aimed to investigate the association between heme, non-heme and total iron intake and risk of GC in Iran.

**Methods:**

In a hospital-based case–control study, nutritionists interviewed 178 pathologically confirmed GC patients and 276 controls using a valid Diet History Questionnaire. Multiple logistic regression model was used to estimate Odds Ratios (OR) and 95% Confidence Intervals (CIs) for iron intake and risk of GC.

**Results:**

Subjects in the highest tertile of total iron intake were 46% less likely to get GC than those in the lowest (OR = 0.54, 95% CI: 0.32–0.92), however, the associations were not significant for intake of heme and non-heme iron. The risk of GC in the highest tertile of total meat intake was 2.51 times higher than the lowest. We found significant associations between GC and chicken (OR = 2.95; 95% CI: 1.66–5.22) and fish intake (OR = 1.89; 95% CI: 1.09–3.27), However, we found no associations between the risk of GC and intake of red meat, salted fish, and liver.

**Conclusion:**

Total iron intake was associated with a lower risk of GC which could be partly due to the high prevalence of anemia in Iran. Although, we could not find any significant association between the risk of GC and the intake of heme and non-hem iron among the Iranian population.

## Introduction

Iron is a vital mineral for various physiological functions in the human body including DNA synthesis, proliferation, cell cycle regulation, and the function of proteins containing iron-sulfur clusters and as well as many redox processes [1]. Although a low level of iron intake is essential for our health, excess iron can cause tissue damage through pro-oxidative effects, potentiating the development of many diseases such as cancer through the generation of reactive oxidative species like peroxides, superoxide, hydroxyl radical, singlet oxygen, and alpha-oxygen [2, 3]. The association between iron intake and different cancers like colorectal and breast has been previously reported [2, 4–6].

Cancer is the second leading cause of death globally and was responsible for 9.6 million deaths in 2018 [7]. Gastric cancer (GC) is the first common cancer in Iranian men with an age-standardized incidence rate (ASR) of 21.6 per 100.000 [8]. Gastric cancer (GC) is a multi-factorial disease. Both environmental and genetic factors play a significant role in the etiology of GC [8, 9].

In the literature to date, the relationship between meats, iron and heme iron intake and GC has been evaluated, but the results are often contradictory and inconsistent [10–13]. The two major forms of iron in the diet are heme and non-heme, both of which are found in several foods. Heme iron is mainly of animal origin, while non-heme iron may also be found in plant foods, fortified foods, or in supplement forms. It has been suggested that heme iron may lead to cancer, but the results are conflicting [13–15]. To the best of our knowledge, there is no study to evaluate the role of heme and non-heme iron intake in GC in Asia, including Iran, where the incidence rate of gastric cancer is considerably high. The incidence of gastric cancer is variable by region and culture [16]. Previous studies were conducted in western countries, but the situation is different in low and middle-income countries because dietary patterns are different and infection of H.pylori is higher, so it is important to replicate these studies in these populations. Also, the prevalence of iron deficiency is high in Iran [17].

In the present study, we studied the association of heme iron and its dietary sources in the Iranian diet and the risk of GC. In addition, we investigated the association of total Iron and non-heme Iron intakes and GC exclusively.

## Material and methods

Data were derived from a hospital-based case–control study conducted at the Cancer Institute of Iran between May 2012 and June 2014. In brief, cases were 178 histopathologically confirmed incident GC patients (132 males and 46 females) aged 40 years or older and were diagnosed maximum one prior to the interview. Patients were admitted to the Cancer Institute of Iran, Imam Khomeini Hospital Complex, which is a referral center admitting patients from all parts of Iran [18].

Controls were 276 healthy individuals (176 males and 100 females) visiting their relatives in the hospitals. We excluded the visitors who were visiting the cancer patients to avoid potential bias due to the shared environment. Every participant signed a written informed consent after the face-to-face description of the study protocol and aims. The study protocol was reviewed and approved by the Ethical Committee of Tehran University of Medical Sciences (No. 17198).

### Covariate assessment

Demographic and general information such as education, anthropometric measures, and selected lifestyle habits including tobacco smoking, alcohol consumption, and personal medical history were collected through a structured questionnaire Venus blood (10 cc) was collected from all participants to determine *H. pylori* seropositivity using IgG antibody.

Body Mass Index (BMI) was calculated as weight in kilograms divided by height in squared meters (kg/m^2^). Since GC causes extensive weight loss, we asked the subjects about weight one year before the interview rather than measuring their weight at the interview. Alcohol intake was asked in DHQ and was converted to grams/day of alcoholic drinks.

### Nutritional assessment

Trained dietitians conducted face-to-face interviews to complete the Persian version of the Diet History Questionnaire (DHQ), which has been validated before [19]. Briefly, it included 146 questions related to the past 12 months’ consumption of foods and Iranian mixed dishes. Patients with GC were requested to recall and report their food intakes before the appearance of cancer symptoms and the controls were asked to report their intake in the year before the interview.

Daily food intakes were used to calculate energy and nutrient intakes based on Food Composition Table in Access software. The Iranian Food Composition Table covers only raw foods and limited nutrients (20). Therefore, we used the McCance and Widdowson's Food Composition Table (21, 22) and supplemented it with Iranian items for some special Iranian foods (20). Consumption of total meat (red meat, fish, and chicken), red meat (beef and lamb meat), chicken, and different types of fish and liver were assessed.

To estimate the heme iron content of foods, we used published information on measured values in different types of meat [20]. The intake of heme iron was calculated by multiplying the estimated heme iron by the mean daily intake of related food sources for each subject.

### Statistical analysis

We excluded 3 subjects who were outliers for the total energy intake from this analysis. We categorized participants into tertiles according to intake of red meat, chicken, fish, liver, total iron, non-heme, and heme iron. The lowest level of consumption (first tertile) was considered as the reference group in the regression models.

Unconditional multiple logistic regression models were used to estimate ORs and corresponding 95% CIs as the measure of associations between intake of heme, non-heme, and total iron and different types of meat intake with GC. We used two regression models including model A, adjusted for age, sex, and energy; and model B in which we adjusted for several confounding variables including age, sex, education, smoking, alcohol intake, *H. pylori* infection and BMI. We used Stata statistical software for analyses (Stata Ver. 14, State Corp., College Station, Texas, USA).

## Results

Demographic and nutritional characteristics of the study participants across tertile categories of heme iron intake are provided in Table 1. There were significant differences in energy, between the subjects in different tertiles of heme Iron in both groups (*p*-value =  < 0.0001).

**Table 1:** Characteristic of study participants according to tertiles of heme Iron intake in a hospital-based case–control study of gastric cancer in Iran (2010 to 2012)

	**Cases**					**Controls**			
**Characteristics**	**T1**	**T2**	**T3**	***p*-value**	**T1**		**T2**	**T3**	***p*-value**
**Number (%)**	59 (33.1)	60 (33.8)	59 (33.1)	0.99	91 (33.3)		92 (33.7)	90 (33.0)	0.99
**Age** (year) Mean (SD)	63.29±11.71	57.25±12.20	62.00±11.43	0.38	53.67±13.03		54.22±10.88	51.88±11.79	0.014*
**Energy **(Kcal) Mean (SD)	2341.83±942.99	2656.99±1050.65	3564.07±1365.59	<0.0001	2399.89±1143.55		2696.64±1150.49	3122.49±1168.00	<0.0001*
**Alcohol drink **(g/d)	1.91±13.40	0.01±0.14	20.40±156.21	0.85	1.22±7.88		1.85±14.82	2.20±12.43	0.40
**BMI **(kg/m^2^) Mean (SD)	25.80±4.18	28.26±18.41	29.48±21.54	0.47	25.93±5.23		26.46±12.04	25.60±5.72	0.78
**Gender** (Male, %)	44 (74.6)	47 (78.3)	41 (69.5)	0.54	56 (61.5)		63 (68.5)	54 (60)	0.44
***H-pylori*** (positive, %)	22 (37.3)	20 (33.3)	26 (44.1)	0.47	57 (62.6)		50 (54.3)	47 (52.2)	0.32
**Marital status** (Married, %)	58 (98.3)	59(98.3)	57 (96.6)	0.32	75 (82.4)		80 (87)	81 (90)	0.84
**Education** (illiterate, %)	36 (61)	34 (56.7)	41 (69.5)	0.34	25 (27.5)		25 (27.2)	22 (24.4)	0.87
**Smoking** (yes %)	37 (62.7)	31 (51.7)	29 (49.2)	0.29	65 (71.4)		61 (66.3)	65 (72.2)	0.63

*p*-values for qualitative variables are resulted from Chi square Test

*p-values for quantitative variables are resulted from Independent T-test

Patients with GC were slightly older (60.83 νs. 53.26 years, *P* =  < 0.0001), had higher BMI (27.81 νs. 26.00 kg/m^2^, *P* = 0.12). They were less likely to be educated (37.6 νs. 73.6%, *P* < 0.0001), more married (97.8 vs. 86.4%, *P* < 0.0001), and drink alcohol (7.44 vs. 1.76 g/d, *P* = 0.40) than controls (data are not shown). Patients also had a lower intake of red meat and protein. The mean heme iron intake of participants was 0.60 ± 0.45 (0.61 ± 0.43 in cases and 0.60 ± 0.47 in controls). According to our results, participants consumed chicken more than red meat and fish respectively.

We found no significant association between GC and dietary intake in grams per day (Table 2). Non-hem and total iron intake decreased and salted fish and heme iron intake increased the risk of GC, however, none of them was significant.

**Table 2:** OR for the association of different types of meat and Iron intake with gastric cancer in a case–control study of gastric cancer in Iran (2010 to 2012)

**Variables**	**Case *N* = **178	**Control *N* = **273	**Adjusted OR** (model A)	**Adjusted OR** (model B)
**Total meat** (g/day)	52.75 ± 33.09	49.55 ± 37.87	1.00 (0.99–1.00)	1.00 (0.99–1.00)
**Red meat** (g/day)	15.98 ± 17.46	17.72 ± 21.5	0.99 (0.98–1.00)	0.99 (0.98–1.00)
**Chicken** (g/day)	26.99 ± 25.68	22.65 ± 25.19	1.00 (0.99–1.01)	1.00 (0.99–1.01)
**Fish** (g/day)	9.76 ± 6.74	9.18 ± 7.4	1.01 (0.98–1.04)	1.00 (0.97–1.03)
**Salted fish** (g/day)	0.41 ± 1.31	0.24 ± 1.57	1.07 (0.93–1.23)	1.05 (0.90–1.22)
**Canned fish** (g/day)	3.85 ± 5.90	3.44 ± 6.40	1.01 (0.98–1.04)	1.01 (0.97–1.04)
**Liver** (g/day)	0.72 ± 1.29	0.69 ± 1.35	1.02 (0.87–1.19)	1.02 (0.86–1.21)
**Heme Iron** (mg/day)	0.61 ± 0.43	0.60 ± 0.47	1.05 (0.66–1.69)	1.03 (0.62–1.69)
**Non-Heme Iron** (mg/day)	19.39 ± 12.13	19.71 ± 12.33	0.98 (0.95–1.00)	0.97 (0.95–1.00)
**Total Iron** (mg/day)	20.00 ± 12.27	20.30 ± 12.42	0.98 (0.95–1.00)	0.97 (0.95–1.00)

A: Adjusted for energy, age and sex

B: Adjusted for age, sex, education, smoking, alcohol, *H. pylori* infection, BMI

After adjustment for all confounding variables, we found a significantly increased risk of GC and intake of total meat (OR = 2.51, 95% CI: 1.20–5.22), chicken (OR = 2.95, 95% CI: 1.66–5.22), and fish (OR = 1.89, 95% CI: 1.09–3.27) intakes in the highest compared to the lowest tertiles (Table 3).

**Table 3:** OR and CI for the association between levels of different types of meat and Iron intake with gastric cancer in a case–control study of gastric cancer in Iran (2010 to 2012)

Variables	T1	T2	T3	*p*-trend
**Total meat**				
Model A (95% CI)	reference	1.52 (0.73–3.15)	2.35 (1.18–4.69)	0.005*
Model B (95% CI)	reference	1.82 (0.83–3.98)	2.51 (1.20–5.22)	0.01*
**Red meat**				
Model A (95% CI)	reference	0.63 (0.38–1.04)	0.83 (0.52–1.32)	0.60
Model B (95% CI)	reference	0.69 (0.40–1.19)	0.72 (0.43–1.20)	0.27
**Chicken**				
Model A (95% CI)	reference	1.92 (1.13–3.27)	2.46 (1.45–4.14)	0.001*
Model B (95% CI)	reference	2.54 (1.41–4.56)	2.95 (1.66–5.22)	0.001*
**Fish**				
Model A (95% CI)	reference	1.55 (0.93–2.61)	1.93 (1.16–3.20)	0.01*
Model B (95% CI)	reference	1.68 (0.96–2.95)	1.89 (1.09–3.27)	0.03*
**Salted fish**				
Model A (95% CI)	reference	1.13(0.70–1.82)	0.72 (0.43–1.19)	0.25
Model B (95% CI)	reference	1.72 (0.86–3.46)	1.15 (0.53–2.46)	0.94
**Canned fish**				
Model A (95% CI)	reference	0.66 (0.39–1.14)	1.57 (0.98–2.51)	0.007*
Model B (95% CI)	reference	0.70 (0.39–1. 26)	1.64 (0.99–2.73)	0.01*
**liver**				
Model A (95% CI)	reference	1.50 (0.92–2.44)	1.14 (0.70–1.86)	0.93
Model B (95% CI)	reference	1.34 (0.78–2.28)	0.99 (0.58–1.68)	0.71
**Heme Iron**				
Model A (95% CI)	reference	0.88 (0.54–1.43)	0.90 (0.55–1.46)	0.68
Model B (95% CI)	reference	0.93 (0.54–1.61)	0.90 (0.53–1.54)	0.72
**Non-Heme Iron**				
Model A (95% CI)	reference	0.61 (0.37–0.99)	0.62 (0.38–1.01)	0.06
Model B (95% CI)	reference	0.62 (0.35–1.08)	0.60 (0.35–1.02)	0.07
**Total Iron**				
Model A (95% CI)	reference	0.59 (0.36–0.96)	0.55 (0.34–0.90)	0.01*
Model B (95% CI)	reference	0.60 (0.34–1.04)	0.54 (0.32–0.92)	0.02*

A: Adjusted for energy, age and sex

B: Adjusted for age, sex, education, smoking, alcohol, *H. pylori* infection, BMI

After adjustment for all confounding variables, we found a lower risk of GC in the highest tertile of the total iron intake compared to the lowest tertile (OR = 0.54, 95% CI: 0.32–0.92, P trend = 0.02). Albeit the associations were not statistically significant for heme (P trend = 0.72) and nonheme (P trend = 0.07) iron.

## Discussion

In this hospital-based case–control study, we found that gastric cancer was directly associated with total meat, chicken and fish intake. The risk of GC decreased with an increase in the intake of total Iron. However, we found no associations between the risk of GC and intake of heme and non-heme iron, red meat, salted fish and liver.

The main strength of our study is the calculation of heme iron values based on Food Tables, while most of the previous studies applied a fixed value of 40% in meats to estimate heme Iron values [21]. We adjusted the associations for different confounding variables including *H. pylori* infection that is an important risk factor for GC and may affect the absorption of nutrients including iron. In addition, we used DHQ, which is a dish-based questionnaire to collect nutrition data and allows estimation of the intake of different types of meat. However, our study had some limitations. The inability to examine risk by sub-type of GC (cardia and non-cardia) due to the small sample size is a potential limitation. Non-cardia GC is more prevalent in Asia and different nutritional risk factors have different effects in relation to cardia and non-cardia GC [22]. In addition, the association between non-heme iron and risk of GC was borderline and the P for trend was 0.07 in the multiple models. A larger sample size is required to evaluate the association of heme and non-heme and the risk of GC in the future. Also, we used the amounts of iron in animal products commonly consumed in Thailand because we did not have these amounts specific for Iranian foods and due to differences in cooking methods and types of meat and this is another limitation of the present study. We did not collect data completely on the cooking methods of different foods including fish and chicken. Further studies should collect more details about the foods and cooking methods.

Previous studies on the association between meat consumption and GC risk showed inconsistent results. In agreement with Larsson [23], we did not find a significant association between the intake of red meat and the risk of GC, while others reported a positive association [9, 12]. World Cancer Research Fund recommends red meat consumption of 500 g/week or lower [24]. We used DHQ to collect dietary information. Although DHQ cannot estimate the intakes of subjects perfectly, the mean intake of red meat in the present study was almost 16 g per day, however, the variation was wide. The socioeconomic status (SES) of subjects affects red meat consumption. Subjects in the high socioeconomic status group eat more red meat than the low SES group [25, 26]. The latest Iranian household food survey in 2002 showed that they eat around 29 g/day of red meat [27], indicating that the consumption of red meat was low in this population and could not affect the cancer risk.

It has been suggested that different chemicals in red meat can cause cancer; including N-nitroso-compounds, Poly-cyclic Aromatic Hydrocarbons, and Hetero Cyclic Aromatic Amines (HCA) [28, 29]. These compounds may appear in some meat processing procedures such as frying, smoking, and barbecuing [30]. In addition, red meat is often considered a high-fat food, which can increase the risk of cancer in humans.

To our surprise, fish intake increased the risk of GC in our study. The results for fish consumption in our study are contradictory to previous results [9, 31, 32]. Munoz reported a decreased risk of GC by fish intake [33], while others did not find significant associations [11, 23]. Based on a meta-analysis, there is no consensus on the role of fish intake in GC [34]. It is worth mentioning that, some studies considered fish in white meat category and did not analyze it separately. As most Iranians consume fish in the fried form, HCA in the fish and also using a high amount of oil, full of hydrogenated fatty acids for frying may lead to carcinogenicity among fish consumers. In addition, fish is subject to the accumulation of toxins and carcinogenic heavy metals or organic compounds which are found in sea or river water [35, 36]. The mean consumption of fish is higher in the north and south of Iran, the shores of the Caspian Sea and the Persian Gulf. However, based on personal communications, food preparation methods vary in the northern and southern parts of the country. While people in the northern part of Iran usually fry the fish, inhabitants in the southern part of Iran use the fish in fried, roasted, grilled, boiled, and stew forms. Besides, people in the northern and central parts of Iran consume river fish that are grown in the pools. However, in the southern part, the fishes come from the Persian Gulf with a wide variety and grow naturally in the sea [37].

We also observed a significant association between chicken intake and the risk of GC. Some studies reported that white meat increases the risk of colorectal cancer [38], while others reported no significant association between poultry intake and GC [23, 39]. Others reported a protective effect of white meat intake [32, 40]. In a meta-analysis by Kim et al., white meat consumption reduced the risk of GC [31]. The protective effect of white meat is related to lesser heme iron content and rich in poly-unsaturated omega-3 fatty acids. The omega-3 content of chicken depends on the type of feed, so it may vary among different countries. Another reason for different results by studies is the definition of white meat intake. Some of them have just reported poultry and chicken as white meat. While, poultry, chicken, duck, and turkey are included in this category. In the present study, we considered only chicken intake. It should be noted that the cooking methods of chicken vary in different regions. Further studies are required to confirm these findings and investigate the reason behind fish and chicken intake and the risk of GC. In addition, analysis of different chemicals in the fish and chicken may provide some clues on the potential carcinogenic effect of these meats among the Iranian population.

Although we found a significant association between total iron intake and a decrease in the risk of GC, we found no associations with heme and non-heme iron. A few studies have been carried out to assess the relationship between iron intake and the risk of GC [10, 11, 13]. Jakszyn et al. in a nested case–control study declared a statistically significant association between heme iron intake and GC risk among European people [11], while Cross et al. reported no significant association between heme intake and cardia or non-cardia adenocarcinoma in a cohort study in the U.S. [13]. Pra et al. reported an increased risk of GI cancers among subjects with low iron intake [41] and Harrison et al. illustrated that iron intake was inversely associated with the intestinal type of GC [42], however, other researchers reported opposite results [11, 14]. According to results in Tables 1 and 2, it is obvious that total meat and total iron intake are lower in our population than in western countries and consumption dispersion is high. The majority of total iron in the present study is derived from non-heme iron which is available in grains and legumes which has a protective effect against GC [43]. Also, when we decompose the total iron to heme and non-heme iron, the difference between cases and controls decreases and we need a larger sample size to have truth justification.

Iron deficiency and anemia increase oxidative stress and DNA damage, which might increase the risk of carcinogenesis, especially in the GI tract [12], through the impairment of several iron-dependent metabolic functions which are related to genome protection and maintenance including immune responses against cancer-initiated cells, metabolism of toxic compounds and redox regulation of DNA biosynthesis and repair [12, 44].

The prevalence of anemia is 20–30% among the Iranian population [45, 46]. The intake of Iron replenishes body stores and may not have pro-oxidant activity. Moreover, gene polymorphisms have an important role in the absorption and metabolism of Iron [47].

## Conclusion

We conclude that total iron intake decreases the risk of GC. However, we did not find a significant association between the risk of GC and the use of heme and non-heme iron. We observed positive associations between GC and intakes of total meat, chicken, and fish. Findings from this study highlight the role of diet in the risk of GC among the Iranian population, where GC is the most common cancer among males and more than 10,000 patients die from this disease. Future studies should be large enough and collect more details about food items and cooking methods.

## Data Availability

The datasets used and/or analyzed during the current study are available from the corresponding author on reasonable request.
